# Field efficacy of a combination of afoxolaner, moxidectin and pyrantel pamoate against natural infestation with *Otodectes cynotis* in dogs

**DOI:** 10.1051/parasite/2026015

**Published:** 2026-03-19

**Authors:** Eric Tielemans, Georgios Sioutas, Elias Papadopoulos

**Affiliations:** 1 Boehringer Ingelheim Animal Health 29 avenue Tony Garnier 69007 Lyon France; 2 Laboratory of Parasitology and Parasitic Diseases, School of Veterinary Medicine, Faculty of Health Sciences, Aristotle University of Thessaloniki 54124 Thessaloniki Greece

**Keywords:** Afoxolaner, Efficacy, NexGard^®^ PLUS, *Otodectes cynotis*, Natural infestation, Dog

## Abstract

*Otodectes cynotis* is the agent of otodectic mange, a disease affecting wild and domestic felines, canines and mustelids. It is a highly contagious and pruritic condition and a major cause of otitis externa in dogs. This study was designed to verify the efficacy of NexGard^®^ PLUS, a combination of afoxolaner, moxidectin and pyrantel pamoate, against natural *O. cynotis* infestations in dogs. It was a blinded, randomised, single-centre, negative controlled clinical efficacy study. Twenty-four naturally infested dogs were allocated to three groups of eight dogs: an untreated control group, a group treated on Day 0 and a group treated on Days 0 and 29 (both groups were treated per label recommendations). Otoscopic examinations were performed on Days -3, 14, 29, 42 and 55 for clinical otodectic mange evaluation. Ear canal flushings were performed on Day 56 for *O. cynotis* counts, the primary variable of efficacy, calculated per comparison of live mites in the treated groups, with the untreated control group. The otoscopic examinations revealed treatment effect by Day 14 and a highly significant effect by Day 29, while the conditions remained unchanged or worsened in the untreated control group. The *O. cynotis* counts revealed reductions of 100% in the group treated once on Day 0 (*p* = 0.0004) and 99.7% (*p* = 0.0006) in the group treated on Days 0 and 29, while 48.8 (geometric mean) live mites were collected in the untreated control group. NexGard^®^ PLUS was demonstrated to be highly efficacious in dogs naturally infested with *O. cynotis* following one or two treatments.

## Abstract (fr)

*Otodectes cynotis* est l’agent de la gale otodectique, une maladie affectant les félins, les canidés et les mustélidés, sauvages et domestiques. Très contagieuse et prurigineuse, cette affection est une cause majeure d’otite externe chez le chien. Cette étude visait à évaluer l’efficacité de NexGard^®^ PLUS, une association d’afoxolaner, de moxidectine et de pamoate de pyrantel, contre les infestations naturelles par *O. cynotis* chez le chien. Il s’agissait d’une étude clinique d’efficacité, monocentrique, randomisée, en double aveugle et contrôlée par un groupe négatif. Vingt-quatre chiens naturellement infestés ont été répartis en trois groupes de huit : un groupe témoin non traité, un groupe traité au jour 0 et un groupe traité aux jours 0 et 29 (les deux derniers groupes ont été traités conformément aux recommandations de l’AMM). Des examens otoscopiques ont été réalisés aux jours -3, 14, 29, 42 et 55 pour l’évaluation clinique de la gale otodectique. Des lavages du conduit auditif ont été effectués au jour 56 pour le dénombrement d’*O. cynotis*, principal critère d’efficacité, calculé par comparaison du nombre d’acariens vivants dans les groupes traités avec celui du groupe témoin non traité. Les examens otoscopiques ont révélé un effet du traitement dès le jour 14 et un effet hautement significatif au jour 29, tandis que l’état des chiens du groupe témoin non traité est resté inchangé ou s’est aggravé. Le dénombrement d’*O. cynotis* a montré une réduction de 100 % dans le groupe traité une seule fois au jour 0 (*p* = 0,0004) et de 99,7 % (*p* = 0,0006) dans le groupe traité aux jours 0 et 29, tandis que 48,8 acariens vivants (moyenne géométrique) ont été collectés dans le groupe témoin non traité. NexGard^®^ PLUS s’est avéré très efficace chez les chiens naturellement infestés par *O. cynotis* après un ou deux traitements.

## Introduction

*Otodectes cynotis* (Hering, 1838) (Acari: Psoroptidae), commonly called “ear mite” is the agent of otocariosis, a highly contagious and pruritic condition affecting felines, canines and mustelids, worldwide [4, 9, 12]. These mites live on the surface of the inner ear canal, but in cases of high level infestation, they can also be found on the outer ear canal and on the epidermis of other body parts, *e.g.* ear pinnae and face [3, 23]. These non-burrowing mites feed on epithelial cells and inflammatory fluids, *e.g.* debris, cells, exudate, lymph and blood [3, 14, 19]. Their five-stage life cycle (egg, larva, protonymph, deutonymph, adult) fully occurs on the host in approximately three weeks. Adults can survive for two months in the host’s ear canal [7]. Transmission occurs through direct contact between animals or fomites, and puppies can become infested during suckling [3, 7, 11, 21]. Outside the host, the mites can survive for a few weeks, but are susceptible to certain environmental conditions such as desiccation, and their infectivity off-the-host is hypothesised to have a duration of 3–4 days [15]. The mites are easily transmitted between cats and dogs. *Otodectes cynotis* is not considered zoonotic, occasional transmission to humans has been reported, but without establishment and breeding cycle [27]. Young animals are more frequently affected than adults [3, 11]. Cats are considered the main reservoir host among domestic animals [20] and foxes are the main reservoir in rural environments [6]. *Otodectes cynotis* is a major cause of otitis externa in dogs, and its main cause in cats [5, 8, 23].

NexGard^®^ PLUS (Boehringer Ingelheim Animal Health) is an oral endectoparasiticide chewable tablet for dogs combining three active ingredients: moxidectin, pyrantel pamoate and afoxolaner. Moxidectin, a macrocyclic lactone compound, is included in this product for its systemic nematicidal activity (namely for the prevention of heartworm disease) [13, 19]; as dosed in the product, it has negligible effect on arthropods (unpublished internal data). Pyrantel pamoate is a tetrahydropyrimidine nematicidal compound, which following oral administration, remains at high concentrations in the digestive tube lumen where it kills the intestinal stages of roundworms and hookworms [18]; it is poorly absorbed through the gastrointestinal mucosa and has no effects on arthropods. Afoxolaner is an isoxazoline insecticide/acaricide compound that has a proven efficacy against fleas, ticks, flying insects and mites [1, 2, 10, 17, 24, 28]. Afoxolaner is dosed at 2.4–5.2 mg/kg (1.14–2.34 mg/lbs) in NexGard^®^ PLUS. Afoxolaner has been used for many years in comparable doses but in different formulations, *i.e.* NexGard^®^ (afoxolaner) and NexGard SPECTRA^®^ (combination of afoxolaner and milbemycin oxime), and was demonstrated to be efficacious in dogs against *O. cynotis* in laboratory and field settings [6, 16, 22, 25].

The objectives of the study described in this manuscript were to verify the efficacy of afoxolaner, when provided in NexGard^®^ PLUS, against *O. cynotis* in field conditions, in naturally infested dogs, and also to compare the efficacy of the product one or two months after administration, as it is unclear whether any surviving stage of the parasite after one month may be able to start a new cycle and re-establish an infestation.

## Materials and methods

### Ethics statement

Animals were managed with due regard for their wellbeing and the study designs and procedures were reviewed and approved by the Ethics Committee of the Aristotle University of Thessaloniki (162009/2024), Greece. The importation and use of NexGard^®^ PLUS was approved by the Greek authorities (National Organisation for Medicine, 84506/2024).

### Study design

This study, conducted from November 2024 to January 2025 in Greece, was a blinded, randomised, single centre, negative-controlled field study with a parallel group design. It was conducted in compliance with VICH GL9 “Good Clinical Practice” and European Union animal welfare directive 2010/63/EU guidelines.

Twenty-four healthy adult mongrel dogs, 14 males and 10 females, weighing 16–29 kg and naturally infested with *Otodectes* were selected for the study. The dogs were hunting dogs living in a rural area. The dogs did not take part in any hunting session during the study, and otherwise were kept in their usual routine, environment and husbandry conditions. All study activities were performed in their kennel by the visiting study personnel.

The 24 dogs were recruited on Day -3, on the basis of health, adequate behaviour and positive diagnosis of otocariosis by otoscopic examination. The dogs were assigned to their study group according to a complete randomised block design. Three study groups of eight dogs each were constituted: Group 1 untreated control, Group 2 treated with NexGard^®^ PLUS investigational veterinary product (IVP) on Day 0, and Group 3 treated with IVP on Days 0 and 29. Each IVP treatment was administered by unmasked personnel per label recommendations. On Day 56, all dogs were sedated for ear canal flushing, mite collection and mite count. Otoscopic evaluations were also performed every two weeks by masked personnel. All dogs were treated with afoxolaner (NexGard^®^) after ear canal flushing, at study completion.

Dogs and their enclosure were observed 1 hour after each treatment to check if any IVP had been vomited. Dogs were observed for health at least once daily by the owner who was instructed to contact the Investigator in case of any abnormality, and every two weeks by the visiting study personnel.

### Efficacy variables

#### Ear mite counts

The primary efficacy variable was live ear mite count obtained on Day 56 from ear canal flushing and content collection. The ear flushing was performed under sedation (dexmedetomidine hydrochloride intramuscularly (IM), 20 μg/kg, reversed with atipamezole IM, 20 μg/kg when the procedure was completed). The ear ducts were filled with paraffin oil and massaged lightly externally until sufficient melting of the ear duct content. The liquid was then collected and drained into a 38 μm sieve. The sieve was rinsed with clean water immediately and the contents of the left and right ear were collected. Both ears were otoscopically examined and if persistent cerumen deposits and/or mites were observed, the flushing procedure was repeated until both ear ducts were clean. The collected materials were examined under a microscope. The mite counts included, for each dog, the addition of all stages (adults, nymphs and larvae) of live *O. cynotis* in both ears, observed under the microscope. Entire and normal looking ear mites (excluding desiccated or fragmented mites) were recorded as live.

#### Otoscopic evaluations

The secondary efficacy variable was a semi-quantitative ear mite burden evaluation and inflammation evaluation obtained by otoscopic examinations performed on Days 14, 29, 42 and 55. Scoring systems were used for the presence of mites (0 = negative, 1 = low infestation, 2 = moderate infestation, 3 = high infestation) and inflammatory signs (0 = negative, 1 = slight debris/cerumen, 2 = moderate debris/cerumen, 3 = marked debris/cerumen).

### Data analysis

The experimental unit was the individual dog.

The efficacy was calculated for each group according to the formula:

Efficacy (%) = 100 × (Mc – Mt) / Mc,

where:

Mc = Geometric or arithmetic mean number of live mites* in dogs in the negative control group (Group 1).

Mt = Geometric or arithmetic mean number of live mites* in dogs in the IVP groups (Group 2 or 3).

*sum of adults, nymphs and larvae, sum of both ears collected from ear flushing on Day 56.

The log-counts (count + 1) of live mites of the treated groups (Groups 2 and 3) were compared to the log-counts (count + 1) of the control group (Group 1), using a mixed analysis of variance model to confirm the miticidal efficacy results. Fixed factors in the model were the group (1–3) and sex (male and female). The block to which each animal was assigned for randomisation was included as a random effect.

If the mite counts did not follow a normal distribution, a Wilcoxon–Mann–Whitney test was used for group comparisons instead.

SAS Version 9.4 was used for all the statistical analyses.

The level of significance of the formal tests was set at 5%, and all tests were two-sided.

## Results

### Ear mite counts

The efficacy results are presented in Table 1.

**Table 1:** Efficacy results based on *O. cynotis* collection and count on Day 56 following ear canals flushing.

Treatment Group^1^	n	GM (AM) live ear mites^2^	% Efficacy GM (AM)^3^	*p*-value (vs Group 1)^4^
Group 1	8	48.8 (58.8)	NA	NA
Group 2	8	0.0 (0.0)	100 (100)	0.0004
Group 3	8	0.1 (0.3)	99.7 (99.6)	0.0006

1 Group 1 = untreated; Group 2 = treated with NexGard^®^ PLUS (IVP) on Day 0; Group 3 = treated with the IVP on Days 0 and 29.

2 GM = Geometric mean. AM = Arithmetic mean. Live ear mites = sum of live adults, nymphs and larvae in both ears.

3 Efficacy (%) = 100 × (Mc – Mt)/Mc, where: Mc = GM (AM) in Group 1, Mt = GM (AM) in Groups 2 or 3.

4 Wilcoxon–Mann–Whitney test.

A high number of live ear mites (GM 48.8, AM 58.8, min 8, max 112) was collected in the untreated control group (Group 1), on Day 56. Dogs in Group 2 (IVP treatment on Day 0) were completely cleared of mites on Day 56. In Group 3 (IVP treatments on Days 0 and 29), two immature live mites were found in one ear of one dog.

### Otoscopic evaluations

**Figure 1:**
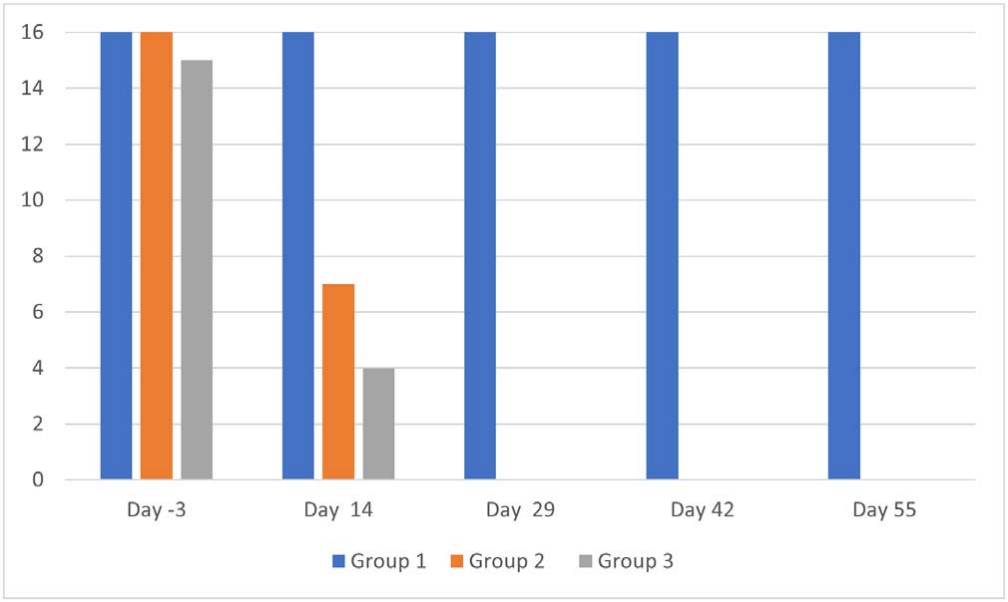
Otoscopic examinations: number of ears per group (maximum possible = 16) positive for live mites. Group 1 = untreated; Group 2 = treated with the IVP on Day 0; Group 3 = treated with the IVP on Days 0 and 29.

**Figure 2:**
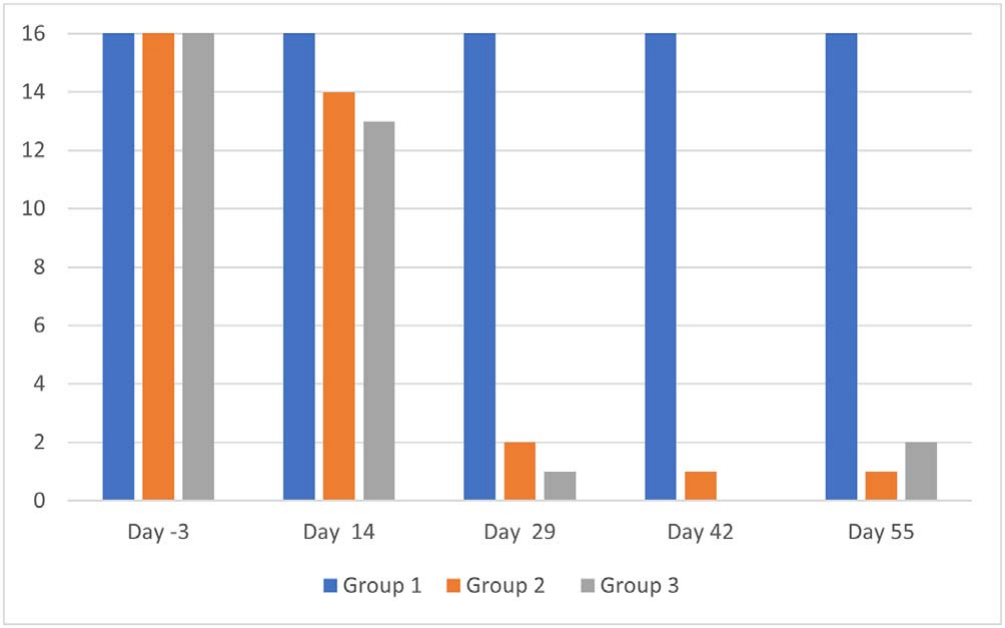
Otoscopic examinations: number of ears per group (maximum possible = 16) positive for debris/cerumen. Group 1 = untreated; Group 2 = treated with the IVP on Day 0; Group 3 = treated with the IVP on Days 0 and 29.

The otoscopic examination results are presented in Table 2. The individual results are not detailed. For clarity, it was preferred to show the sum of scores per group at each assessment time point and for each evaluation criterion. The number of ears with a positive result for each evaluation criterion, but without consideration of the scores, are presented in Figures 1 and 2.

**Table 2:** Otoscopic examination results. Sum of scores per group (maximum possible = 48) at each assessment time point, inclusive of both ears and of the eight dogs in each group.

	Live mites			Debris/cerumen		
Day	Group 1	Group 2	Group 3	Group 1	Group 2	Group 3
-3	18	17	17	22	22	21
14	23	7	4	28	14	14
29	29	0	0	38	2	1
42	30	0	0	36	2	0
55	25	0	0	34	1	2

Scoring system for live mites: 0 = negative, 1 = low infestation, 2 = moderate infestation, 3 = high infestation.

Scoring system for debris/cerumen: 0 = negative, 1 = low level, 2 = moderate level, 3 = high level.

Group 1 = untreated; Group 2 = treated with the IVP on Day 0; Group 3 = treated with the IVP on Days 0 and 29.

At baseline, on Day -3, the three groups were comparable in terms of presence of live mites and inflammatory signs. All dogs in each group were positive for live mites (16, 16 and 15 ears positive in Groups 1, 2 and 3, respectively) and debris/cerumen (all 16 ears were positive in each group).

In the untreated control group, the otocariosis signs remained consistent or worsened until Day 55, as the 8 dogs and 16 ears remained inflamed and infested, with overall increasing scores.

In both IVP treated groups, the otocariosis signs had a comparable course. On Day 14, a moderate reduction in live mites was observed, as 11 in 32 ears were positive for mites, and from Day 29 onwards, no mites were visible (Fig. 1). The debris/cerumen had slightly improved on Day 14 and was then significantly improved from Day 29 onwards (Fig. 2).

### Health observations

No abnormal observations were reported from the owner and visiting personnel. No abnormality (*e.g.*, vomit of IVP) was reported after treatments.

## Discussion

This is the first study verifying the efficacy of afoxolaner administered orally in combination with moxidectin and pyrantel pamoate against *O. cynotis* mite infestation. Afoxolaner differently formulated was previously demonstrated efficacious in experimental and field studies [6, 16, 22, 25]. It is also the first study verifying the efficacy of afoxolaner two months after a single administration.

The ear mite counts performed on Day 56, eight weeks after a single IVP treatment (Group 2), or four weeks after a second IVP treatment (Group 3), and in comparison with the high number of mites found in the untreated control group (Group 1), revealed that the IVP significantly reduced infestation in both treatment regimens. Results also showed that a repeated monthly treatment did not have an advantage *versus* a single treatment, after two months. Group 2 also revealed that after one month (the claimed efficacy duration of the IVP for fleas and ticks), no immature *O. cynotis* was present and able to start a life cycle identifiable at two months. The only *O. cynotis* that were collected on Day 56 in the treated groups were two immature forms in the left ear of one dog that had been treated with the IVP on Days 0 and 29 (the left ear of that dog had been scored negative for ear mite and debris/cerumen during the otoscopic examinations on Days 29, 42 and 55). The authors suggest the following three hypotheses to explain this finding. Inside the ear canal, immature forms (*e.g.* eggs) might be kept unexposed to an active ingredient in wax or cerumen and be released alive in a longer term. Eggs fallen out of the ear after dwelling on the skin and haircoat may have later on hatched and entered the ear canal for development. Nymphs or larvae may have crawled from nearby untreated cages and infested the ear of that dog, confirming that all dogs, cats and mustelids in the same environment should be treated.

The otoscopic examinations performed every two weeks (on Days 14, 29, 42 and 55) revealed that the IVP had a marked effect by Day 14, when the number of ears positive for live mites had dropped from 31 to 11 (inclusive of both Groups 2 and 3), and a maximum effect by Day 29, when the number of ears positive for live mites had dropped from 31 to 0, and remained so on Days 42 and 55 without a second treatment (Group 2) or with a second treatment on Day 29 (Group 3). The IVP effect on debris/cerumen was notable on Day 14 as the cumulated scoring (Table 2) in the treated groups (14 and 14) was half that of the untreated control group (28). This effect was marked on Days 29, 42 and 55 as the cumulative scorings in the treated groups ranged from 0 to 2, while they ranged from 34 to 38 in the untreated control group.

Of note, at baseline examination, 23 of the 24 dogs had positive mite and debris/cerumen observation in both ears, one dog had one ear negative for mites; however, both ears were positive for debris/cerumen. This confirms that most cases of otitis externa caused by *O. cynotis* are bilateral [3, 4, 7].

These observations allowed the conclusion that afoxolaner administered orally in combination with moxidectin and pyrantel pamoate significantly reduces *O. cynotis* infestations after one treatment, that no immature forms started a cycle during a second month after a single treatment, but that it is possible to find small numbers of immature forms in the ear canal for an undefined time.

NexGard^®^ PLUS was demonstrated to be safe and highly efficacious in dogs naturally infested with *O. cynotis* and provides a favourable solution for dogs infested with otodectic mange and in environments where there is a risk of heartworm disease transmission and intestinal nematode infections.
